# It Takes a Village: Optimal Graduate Medical Education Requires a Deliberately Developmental Organization

**DOI:** 10.5334/pme.936

**Published:** 2023-07-28

**Authors:** Kelsey A. Miller, Joshua Nagler, Margaret Wolff, Daniel J. Schumacher, Martin V. Pusic

**Affiliations:** 1 Pediatrics and Emergency Medicine, Harvard Medical School, Boston, MA, USA; 2 Emergency Medicine and Pediatrics, University of Michigan Medical School, Ann Arbor, MI, USA; 3 Cincinnati Children’s Hospital Medical Center and the University of Cincinnati College of Medicine, Cincinnati, OH, USA

## Abstract

Coaching is proposed as a means of improving the learning culture of medicine. By fostering trusting teacher-learner relationships, learners are encouraged to embrace feedback and make the most of failure. This paper posits that a cultural shift is necessary to fully harness the potential of coaching in graduate medical education. We introduce the deliberately developmental organization framework, a conceptual model focusing on three core dimensions: developmental communities, developmental aspirations, and developmental practices. These dimensions broaden the scope of coaching interactions. Implementing this organizational change within graduate medical education might be challenging, yet we argue that embracing deliberately developmental principles can embed coaching into everyday interactions and foster a culture in which discussing failure to maximize learning becomes acceptable. By applying the dimensions of developmental communities, aspirations, and practices, we present a six-principle roadmap towards transforming graduate medical education training programs into deliberately developmental organizations.

## Introduction

Trust and vulnerability promote effective learning cultures, enabling individuals to incorporate feedback and learn from failures [1,2]. These attributes may be elusive in graduate medical education (GME) programs characterized by frequent trainee transitions, the need to balance learning with patient care, and a strong emphasis on assessment [3,4,5,6,7]. Coaching has been proposed as a means of embracing feedback and failure by aligning the teacher and the learner around shared developmental goals and performance improvement plans for the learner [2,8,9,10]. However, current models of coaching in GME may not reach their full potential due to a perception of coaching as confined to limited spaces and interactions. We present a comprehensive model, from the business literature, that underscores the organization’s role in coaching.

Coaching in medical education has been conceptualized as two distinct types: coaching in the moment (performance coaching) and coaching over time (longitudinal coaching) [11]. The former capitalizes on teachable moments in clinical settings amenable to direct observation, where feedback is employed to identify performance objectives and design plans to address them [10]. The brevity of these interactions may not allow for the necessary trust and vulnerability for maximal learning [12,13]. Furthermore, performance coaching necessitates the “teacher” possess the requisite skills to transform feedback into actionable coaching [10,12]. Consequently, medical education has predominately focused on longitudinal coaching by designated coaches. These coaches help learners amalgamate a variety of performance data, identify learning goals, and conceive plans for achieving desired learning [9,11,13,14]. Although these relationships aspire to cultivate an educational alliance and create the trust and safety essential for improvement, they can be hampered by infrequent interactions and the quality of the data received —if direct observation opportunities are limited, coaches may need to rely on evaluations and self-assessment.

Performance and longitudinal coaching are influenced by the surrounding learning culture [2,9]. These relationships require a continuous improvement stance and are subject to potential distortion by the emphasis on assessment within medical training [10,15,16]. Trainees’ awareness of being assessed can promote “performance” postures – impression management focused on appearing competent and protecting one’s reputation [6,7,17,18,19]. This focus on performance within GME organizations can inhibit the frank self-assessment and accurate appraisals by others essential to optimizing any type of coaching.

Boosting the potential of coaching will entail a cultural shift within medical education: a transition from intermittent individual-level coaching interactions to an organization-wide approach that normalizes and encourages the need for development. We present an existing conceptual model for achieving this transformation – the deliberately developmental organization (DDO) [20,21]. At its core, a DDO is one that makes coaching pervasive throughout an organization. It has been implemented in the business world, where DDOs prove to be more optimal incubators for learning than traditional one-on-one coaching models [20,21,22].

In this manuscript, we describe the core principles of a DDO and propose a road map for organizational change to transform GME to incorporate these principles. Recognizing an organization as a collection of individuals, we use “organization” to describe the culture, structure, and processes within which these individuals operate [23].

## Deliberately Developmental Organizations as the Optimal Incubator for Learner Growth

The DDO framework, originated by Robert Kegan and Lisa Lahey, delineates how organizations can foster member development through three mutually reinforcing dimensions: developmental communities, developmental aspirations, and developmental practices (Box 1) [21,24].

**Box 1 Deliberately developmental organization**

Made up of three key dimensions that mutually reinforce each other:

- Developmental community: cultivating a culture where everyone shares responsibility for development and individuals are valued and respected, even at their well-intentioned worst.
- Developmental aspirations: prioritizing growth as fundamental at both individual and organizational levels.
- Developmental practices: designing routine work to highlight and leverage failures for personal and organizational improvement.

The most important departure from traditional workplace organizations is the transformation into a *developmental community* where all individuals bear responsibility for their own and their colleagues’ growth [20]. This community undergirds and is bolstered by the DDO’s other dimensions: supporting individual *developmental aspirations* and employing *developmental practices* to intentionally surface instructive failures for personal and organizational improvement.

The cornerstone of a DDO is the idea that *developmental communities* are integral to both individual and organizational development, making development everyone’s responsibility every day. Traditionally, coaches help individuals self-monitor and assume responsibility for change. Outside the coaching bubble, individuals may spend time hiding, defending, avoiding, or failing to acknowledge gaps and internal tendencies rather than engaging in improvement [20,25]. They may miss opportunities to demonstrate vulnerability for the sake of growth. The DDO extends the coaching paradigm into developmental communities. Growth opportunities may emerge in routine interactions, and a DDO recognizes that everyone needs help identifying and seizing such chances. In a DDO culture, weaknesses can be surfaced and discussed in any interaction as vulnerability is embraced by the organization, and individuals feel respected and worthy, even at their (well-intentioned) worst [20,21]. Without this accepting culture, publicly identifying limitations and learning goals may be challenging.

A DDO also dissolves the distinction between teachers and learners by prioritizing *developmental aspirations* for continuous improvement of all its members. Unlike traditional coaching seen in sports or music, in a DDO every individual is responsible for their own development, for the development of their colleagues, and for contributing to a developmental culture [20,21]. When coaching is confined to individual relationships or moments, it limits the ability to learn from each person’s expertise and may not provide sufficient support for the vulnerability needed to scrutinize weaknesses, self-monitor effectively, or align internal and external assessments. A DDO understands that every individual can teach because people have different strengths and areas of knowledge regardless of their experience level. The required trust is achieved by making everyone partners with the shared expectation that vulnerability leads to identification of blind spots, acceptance of the need to learn, and support for growth.

A DDO supports individuals in pursuing their *developmental aspirations* through *developmental practices* that reframe experiences of incompetency as “desirable difficulties” required to identify blind spots and self-assess accurately [20,21,26]. The structures and practices of a DDO engage people in surfacing and tackling growth opportunities, combating the tendency to hide weakness or avoid challenges to appear competent. The result is the realization of coaching’s key aspects: a focus on goal-oriented individual growth that properly values failure – not as an acceptable final outcome, but as an inevitable and integral part of learning and continuous growth [21,26].

## Reimagining Graduate Medical Education as a DDO: The Challenges

Achieving a DDO culture within GME is indeed daunting, with clear challenges at program, institutional, and national levels. In this section we identify these obstacles so that we can subsequently build a case for organizational change to overcome them.

Healthcare is high stakes. Medical education transpires within programs and organizations whose core mission is safe patient care. Allowing learners to grapple with “desirable difficulties” or fail at these tasks seems opposed to this mission. However, the underpinning of a DDO is the conviction that an organization’s performance is inseparable from its commitment to developing its members [21]. DDOs have been successfully implemented in high pressure businesses such as Bridgewater, an award-winning hedge fund [20,22]. DDOs like Bridgewater do not merely celebrate mistakes. They utilize inevitable errors as opportunities to analyze and learn. Bridgewater requires its employees to log problems and consider how they and their colleagues contribute to suboptimal execution. The goal is to generate an accurate picture of an individual’s ability to eliminate gaps between actual and desired performance. A DDO combines a growth mindset with a continuous commitment to improve oneself and the organization [26]. Allowing trainees to make mistakes as part of their development is already acknowledged in medical education [27]. In these cases, patient safety and trainee learning are often seen as a tradeoff. Although businesses can take a longer-term perspective, delivering suboptimal care to a current patient for the benefit of future patients may seem more ethically fraught. Yet, the presence of trainees already involves “inexpert clinical performance” [27]. What is needed is clear organizational guidance on tolerable failures and how to protect patients from them. Simulation offers potential for implementing a DDO approach in medicine. It allows distribution of mistakes between the clinical and simulated environment, encourages discussion of failure in a safe space, and challenges people at any performance level [28].

A second challenge is the need for widespread coaching skills. If “everyone” is to coach, then “everyone” will need to give constructive feedback, highlight gaps, probe, and engage in collaborative goal-setting [10,29]. Attempting coaching without these skills can undermine trust and damage the credibility of the feedback and coaching [1,4,12,29,30]. However, these competencies can be taught. Already, faculty development programs and feedback frameworks exist to equip health professionals for coaching [12,31,32,33]. The challenge lies in disseminating this training organization-wide, a challenging feat requiring time, resources, and commitment from the organization’s members. Furthermore, if coaching is everyone’s responsibility rather than the duty of a select few, it may become less pervasive. Widespread investment in coaching becomes possible if an organization embeds coaching in core workplace activities. Businesses have achieved this when their leaders commit time to developing employees’ coaching skills and integrating reflection and coaching into daily work routines [20,22].

Another potential barrier to the DDO framework in medicine lies in the emphasis on performance and assessment for patient care and academic advancement. There is a baseline performance level required to progress to the next stage of training, to deliver quality patient care, and to contribute academically to increasing medical knowledge. A necessary focus of GME is assessing the competency of medical trainees [34,35]. Part of the argument for longitudinal coaching relationships is that learners feel safer revealing gaps to individuals uninvolved in assessment. If everyone is a coach, coaching and assessment will overlap. In a DDO, this overlap cannot undermine the developmental community. Sports show that a demarcation between coaching and assessment is not always necessary. Athletic coaches are responsible for developing athletes and deciding who to play. Similarly, fostering a deliberately developmental environment will require assessments-for-learning that balance rewarding current performance, unmasking areas for improvement, and fostering continuous learning [36]. A marriage of coaching and assessment may help eliminate assessments that fail to uncover trainees’ weaknesses and provide an incomplete picture of their competency [36].

The hierarchical culture of medicine presents another obstacle. A DDO blurs the boundary between trainees and faculty by promoting coaching and development for all. Within the physician profession, the training hierarchy impacts who provides feedback to learners and how the feedback is received [15]. Interprofessional boundaries also influence how learners seek and value feedback [37,38]. Examples from the business world show that overcoming power structures is possible by ensuring leaders participate fully in the demands of a DDO and model developmental behavior [20]. Leaders openly work on their weaknesses through daily conversational routines that invite those lower down the hierarchy to challenge them, publicly sharing their performance reviews, and inviting all employees to redesign processes that aren’t working well. For this to work in medicine, faculty and other professionals need to recognize their own needs for feedback and growth [39]. Faculty supervisors– the leaders of GME training – will need to serve as models. And GME leadership may be distributed across multiple “organizations”. Educators seeking to transform their training program into a DDO will want to ensure the encompassing division, institution, and regulatory bodies commit to the change.

Finally, it is important to recognize unique features of medical training that may hinder trust between trainees and the people surrounding them. To become DDOs, businesses invest in helping employees get to know each other to generate the necessary sense of community [20,21,22]. In medical training, however, the actors and contexts are constantly changing. Trainees rotate in and out of clinical spaces, resulting in more transient relationships compared to those seen in music, sports, and business [2,40]. This can limit trust, alter the credibility of feedback, add stress, and increase pressure to perform [1,4,40,41,42]. For these more transient relationships to support a DDO, organizational culture is key. There are educational models to address the disjointed nature of training, such as longitudinal-integrated-clerkships at the undergraduate medical education level [43,44]. Furthermore, trainees are more trusting of individuals they perceive to be invested in their growth and development [19,41,45,46]. If trainees repeatedly experience individuals valuing authenticity and transparency around failure, they can approach new relationships with this expectation rather than building trust from scratch [22].

## Reimagining Graduate Medical Education as a DDO: The Vision

We described the obstacles to reimagining GME as a DDO with the aim of directly addressing them to pave the way for this transformation. Applying the dimensions of developmental communities, aspirations, and practices, we now propose a roadmap toward achieving this change. We suggest six principles for cultivating a DDO culture within GME (Table 1). Our focus is on organizational change, which we approach beginning at the program level.

**Table 1:** Principles for creating a deliberately developmental culture within graduate medical education


PRINCIPLE	DESCRIPTION

Principle 1: Everyone is a coach	Individuals approach routine interactions with each other from a coaching mindset, regardless of whether they are in formal coaching roles

Principle 2: Extraordinarily supportive environment	Optimize support for trainees and faculty who are continuously coached and expected to grow by encouraging the exposure and ownership of gaps

Principle 3: Group-share growth goals	Faculty, trainees, and interprofessional team members share their growth goals regularly

Principle 4: Weaknesses are assets	Failures are reframed as opportunities to be surfaced to maximize learning

Principle 5: Design for desirable difficulties	Place trainees and faculty in situations where they are continually challenged

Principle 6: Scaffold self-reflection	Establish structures and conversational practices to scaffold individuals in self-reflection and taking responsibility for their own improvement


To make these principles concrete and actionable, we illustrate how they lead to changes at the individual, program, institutional, and national levels (Table 2, Supplementary Table).

**Table 2:** Mono, micro, meso, and macro changes required to create a deliberately developmental organization*


DEVELOPMENTAL ASPIRATIONS	

Mono Level	Centering on self-improvement makes growth and future performance more highly valued than current performance and combats tendency to hide weaknesses and errors Regularly identify and share growth goals

Micro Level	Coaches recast failures as opportunities, helping learners to surface and exploit suboptimal performance rather than hide weaknesses

Meso Level	Division/Department: Promote regular sharing of growth goals by faculty and trainees (start of procedure/shift, during conference, etc.) Combat tendency to hide weakness by supporting both self-assessment and disclosure Institution: Prioritize individual growth as central to organizational success through valuing a developmental orientation in recruiting and evaluating learners & establishing structural frameworks and incentives for goal-setting and sharing Create and protect forums for interprofessional colleagues to share growth goals across professional boundaries

Macro Level	Strengthen program requirements around setting and supporting implementation of learning goals Leverage entrustable professional activities and milestones to support programs in building curricular structures that regularly share identified weaknesses with individuals Create requirements around setting improvement goals for maintenance of certification

**DEVELOPMENTAL COMMUNITIES**	

Mono Level	Embrace responsibility for pursuing one’s own growth and for helping others do the same

Micro Level	Approach coaching interactions open to feedback and trusting the other’s commitment to one’s development Faculty and interprofessional colleagues exemplify openness to coaching by showing humility, engaging in intellectual candor, and asking for feedback on self-identified weaknesses and unknown gaps

Meso Level	Division/Department: Coaching not limited to individual relationships: train all faculty and trainees on coaching and being coached Spaces are created for coaching exchanges during clinical care and/or educational routines Leaders are “chief coaches” while demonstrably inviting coaching and visibly working on growing themselves Institution: Optimize support for continuously being coached and asked to grow; this includes role modeling from leadership, protecting and incentivizing coaching, and ensuring a supportive clinical learning environment Facilitate inter-professional coaching: create spaces for exchange of feedback, empower other professions to coach

Macro Level	Competence in coaching treated as essential to the profession and embedded in program requirements/assessments Commitment to coaching reinforced by incorporation in professional organizations’ requirements for certification

**DEVELOPMENTAL PRACTICES**	

Mono Level	Make internal operations public and invite feedback to identify personal tendencies, gaps, and blind spots Assume responsibility for self-reflection and improvement, including searching for and pursuing constructive destabilization (activities resulting in feelings of inadequacy or incompetence, representing opportunities for growth)

Micro Level	Respond to moments of learner vulnerability (sharing internal operations, identifying personal weaknesses) with respect and support, avoiding judgement Faculty encourage trainees to experience constructive destabilization and reframe resulting feelings of incompetence as learning opportunities; faculty pursue their own opportunities for constructive destabilization

Meso Level	Division/Department: Transform clinical and didactic offerings into safe spaces with explicitly stated expectations of non-judgment and a focus on improvement (as is the case in Morbidity & Mortality and quality improvement sessions) Develop conversational routines and activities to 1) engage individuals in reflecting on and sharing personal tendencies and internal thought processes and 2) promote feedback from others in response Use assessment data and competency mapping to design training to promote graduated challenges Institution: Establish expectations that all trainees and professionals adhere to a culture of “well-held vulnerability” Structure training and professional development programs to expose individuals to areas in which less proficient

Macro Level	Allow assessments of competency to inform individualization of training to promote graduated challenges Support programs in using assessments of competency to match gaps to required graduate and continuing medical education and other professional development activities Incorporate self-reflection and sharing feedback into certification and maintenance of certification by professional bodies


* Mono = individual, micro = individual coaching relationship, meso = division/department and/or institution, macro = regulatory bodies.

### Developmental communities in graduate medical education

#### Principle 1: Everyone is a coach

While a primary coach may be identified, individuals throughout the organization ideally perceive coaching as an important responsibility. Coaching becomes an everyday activity, where individuals approach routine interactions from a coaching mindset, irrespective of formal coaching roles. This forms the foundation of the developmental community, essential to support the other dimensions of a DDO. To facilitate this, several organizational changes are required. Both trainees and faculty will benefit from training to develop coaching competencies and a toolbox of coaching tools, such as the CBD (Competence by Design) or R2C2 (relationship, reactions, content, coaching) models [10,31,32,33]. Successful DDOs achieve this by implementing boot camps for new employees or having leaders curate a video- and text-based curriculum to share stories of individual development across the organization (Box 2) [20,22]. Recognizing the hierarchy in medical education, modeling will primarily need to come from faculty, particularly those in leadership. Coaching interactions should not be limited to trainees but should also include supervising faculty who openly identify and work on their growth edge. Leaders – such as program directors or department chairs – become “chief coaches,” responsible for modeling effective coaching and developing coaching skills in others. They emphasize that everyone needs coaching and is expected to contribute to improving others [20,22]. Programs and departments will have to assess coaching competencies and provide additional training to those who need it. Faculty reviews and feedback should incorporate an assessment of each faculty member’s performance as a coach and their contribution to promoting a coaching culture.

**Box 2 Practical suggestions for achieving a DDO culture - Examples from Decurion**

Decurion, a successful entertainment company recognized as an exemplary DDO, provides practical examples of how the DDO framework has been implemented:

- Initiating daily check-ins and check-outs to ensure employees feel connected to colleagues as part of a supportive community (developmental communities) and to scaffold self-reflection (developmental practices)
- Creating visual competency boards to track and publicly display individuals’ capabilities across a spectrum of job competencies, allowing others to understand what individuals are working on (developmental aspirations).
- Using visual competency boards to identify ideal job assignments that maximize individual growth and fulfill the needs of the business (developmental practices)
- Scheduling weekly management meetings to discuss employees’ goals and performance to decide who needs more responsibility (developmental practices)
- Asking employees to identify how their personal growth goals align with business needs during performance reviews (developmental practices)

#### Principle 2: Extraordinarily supportive environment

Programs will need to increase support for the trainees and faculty who are expected to grow through continuous coaching. Identifying and pushing oneself to the limits of one’s competency can be beneficial for growth but may also be psychologically painful and destabilizing. It requires widespread trust and respect to ensure trainees feel part of a developmental community. Creating this safe environment may be particularly challenging for trainees who constantly rotate into new clinical workplaces [44]. It requires repeated positive experiences for trainees who expose gaps in knowledge, skills, or experience. Programs can promote such acceptance by communicating DDO expectations to faculty. Specifically, programs will need to communicate the expectation of continuous growth and outline ways in which faculty members can support the culture of “well-held vulnerability” that such growth necessitates. Bridgewater’s chief executive officer achieved this with company-wide messaging urging employees to focus more on how fast individuals are learning rather than on their current performance. Within medicine, there are models like Morbidity & Mortality conferences and quality improvement efforts, which set clear expectations of non-judgment and a focus on improvement [45,46]. These expectations can be explicitly expanded to clinical and didactic sessions, as well as verbal and written feedback [46]. When leaders become aware of instances where vulnerability is not well-held by faculty or trainees, these will need to be addressed.

Creating this environment necessitates normalizing the sometimes-painful self-reflection and self-exposure required for growth as the experience of trainees *and* faculty. If trainees witness faculty members openly disclosing their own shortcomings, this helps trainees realize that physicians are not less capable or admirable for having done so. The potential for storytelling as a form of modeling by senior members of the medical education community presents itself here. Intellectual candor – faculty expressing their inner struggles – can invite reciprocal vulnerability and build trust [1]. Both trainees and faculty can be rewarded in their assessments for identifying or exposing weaknesses and for assisting others in doing the same.

Through many positive interactions, trainees see struggling is accepted and come to believe everyone in the program is committed to their development. They can become comfortable with disclosing a weakness as soon as it is identified. To facilitate this, the entertainment company Decurion has implemented daily check-ins and check-outs to ensure employees feel connected to colleagues and do not have to wait for specific venues or individuals to discuss their struggles [1].

### Developmental aspirations in graduate medical education

#### Principle 3: Group-share growth goals

DDOs aim to develop practicing physicians and professionals as well as trainees. Programs will need to find ways to help faculty, trainees, and interprofessional team members regularly share their developmental aspirations. When continual improvement is an aspiration, members of the faculty invest in achieving their own developmental goals and in supporting others to do the same. This can be facilitated by structures that promote regular self-reflection on growth goals, as well as allocating time and space for trainees and faculty to share these at the start of a shift, clinic, procedure, or didactic session.

Moreover, the emphasis on development should be interprofessional, with team members across disciplines committed to their own growth and supporting that of others. To create forums for interprofessional sharing, training program leadership will need to collaborate and form alliances with physician and non-physician clinical leaders [47].

Identifying and sharing goals takes time and effort. Trainees, faculty, and healthcare professionals are busy and face competing demands [5,47]. Programs will need to direct individual and organizational resources toward promoting the discussion of developmental aspirations. One way to achieve this is by creating processes for sharing individual learning objectives and assessment data [24]. Decurion uses visual competency boards to track individuals’ capabilities in 15 job competencies, which are publicly displayed to allow others to see what each individual is working on [22]. Another strategy is to recognize participation in goal-sharing when making decisions about protected time, compensation, awards, and promotion. Taking these steps can signal that developmental aspirations are valued by the program and department.

#### Principle 4: Weaknesses are assets

Both faculty and trainees experience moments of suboptimal performance. The goal is to reinterpret these inevitable “failures” as opportunities to address developmental aspirations. This process is easier in medical simulations, which are seen as learning opportunities where mistakes are “safe” and provide valuable material for growth [28]. But learning from mistakes doesn’t need to be confined or hidden inside a simulation center. This language and practice can be applied to other aspects of GME, such as informal and formal clinical feedback, as well as conferences. Faculty members play an important role in modeling this by disclosing their own gaps and highlighting these as opportunities for life-long learning. When developing coaching skills among faculty, programs will need to equip them to help trainees view failures as opportunities and to discourage them from hiding weaknesses. Self-assessment and disclosure for both faculty and trainees can be incorporated into clinical and educational routines. This naturally merges with sharing growth goals, ideally tied to areas where development is needed.

Programs also need to rethink their approach to assessment to avoid discouraging trainees from revealing areas where they may be struggling. Clear communication about when assessment is formative or summative can help make failure feel safer for learners – for example, using words like “coaching” to describe activities aimed at learner development instead of “assessment” when no summative judgments are being made [6,48]. When assessments are necessary, focusing more on how trainees are progressing rather than how they are currently performing can help programs combat the aforementioned “performance postures” [7,19,48]. To create this type of improvement culture, assessments of entrustable professional activities and milestones can be used to regularly share identified weaknesses with trainees [24]. Doing so will help normalize failure as inevitable and stress its vital role in development.

### Developmental practices in graduate medical education

#### Principle 5: Design for desirable difficulties

To further transition towards a DDO culture, training programs can aim to put trainees in situations where they encounter constructive destabilization (activities resulting in feelings of inadequacy or incompetence, representing opportunities for growth) [20]. The general trajectory of medical training includes graduated challenges, and this can be amplified by individualizing the trajectory for each trainee. In DDO vernacular, these situations are termed developmental practices. Assessment data can help programs incorporate developmental practices [24]. Decurion uses its visual competency boards and maps of each employee’s progress to identify ideal job assignments that maximize each individual’s growth and the needs of the business [20,22]. Management teams meet weekly to discuss employees’ goals and performance to decide who needs more responsibility. Using this model, clinical competency committees can move toward more real-time sharing of competency-based assessments and collaboration with program leadership to inform decisions about rotation selection and design.

Programs will also need to ensure faculty are prepared to frame the resulting feelings of incompetency as opportunities for development. For example, mastery can be presented as a fluid rather than a fixed goal, so trainees feel successful even as they are continually challenged [26]. One way to promote this is to adopt the same approach with faculty professional development: expect faculty to seek their own opportunities for constructive destabilization. By doing so, faculty gain credibility when encouraging trainees to view struggle and failure as essential catalysts for growth [1].

#### Principle 6: Scaffold self-reflection

Another important developmental practice is scaffolded (supported) self-reflection. This can help individuals negotiate constructive destabilization, recognizing that it can be an emotionally fraught process. Programs can develop conversational practices to overcome tendencies to avoid conflict or embarrassment, which often go hand-in-hand with the vulnerability associated with deep learning. Whenever possible, these conversations should not only engage individuals in reflecting on personal tendencies and sharing internal thought processes, but also promote non-judgmental feedback from others in response. If these developmental practices are regularly implemented by trainees and faculty, they become less risky. Trainees gradually come to trust that revealing their growth edge or discussing “the undiscussable” leads to positive rather than negative outcomes. The aforementioned check-ins and check-outs represent one way of building a practice of self-reflection [20,22].

In addition, educators can ask trainees to share their reflections. DDOs in the business world ask employees to identify ways their personal growth goals align with business needs during employee reviews [22]. Similarly, programs can encourage trainees to identify opportunities for constructive destabilization themselves. Creating facilitated opportunities for trainees to discuss alignment between internal and external assessments can also reveal blind spots and improve self-reflection [49].

## From Here to There: Gradual Transformation

**Figure 1:**
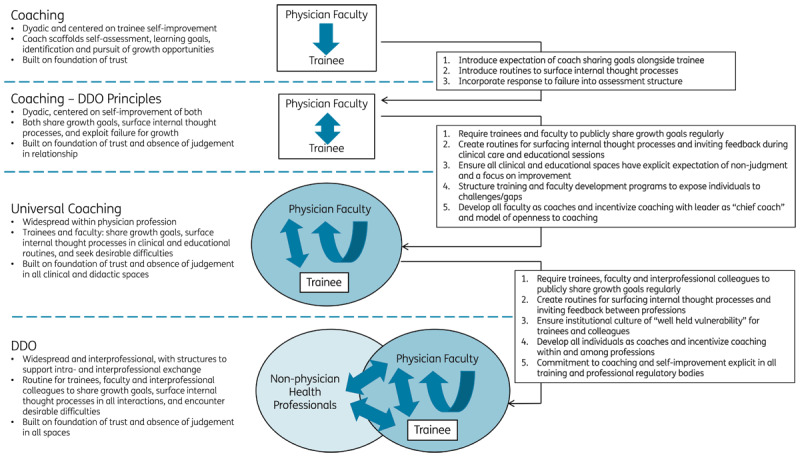
Pathway to a deliberately developmental organization

Healthcare and medical education are complex. They consist of numerous stakeholders and institutions, each with strong traditions and cultures. Unlike business, medical training is characterized by individuals rapidly cycling in and out of diverse contexts and organizations. Imagining a rapid and dramatic establishment of a DDO culture within GME seems unrealistic. Cultural transformation will be challenging– training programs grapple with many immediate and long-standing educational issues, and they operate within complex healthcare organizations over which they have limited control. However, this reality does not negate the value of aspiring towards a DDO culture or starting the process of actualizing it. Indeed, the current emphasis on coaching within medical education provides an excellent springboard for promoting a DDO approach (Figure 1). While organizational change is necessary, this transition needs to be incremental and gradual. A first step involves the continued implementation and expansion of coaching programs, as endorsed by organizations like the American Medical Association and the Royal College of Physicians and Surgeons of Canada [8,11].

While current coaching models have primarily focused on trainee development, an important subsequent step is to reconceptualize them as bidirectional relationships where faculty members share growth goals, discuss internal thought processes, and use failures as opportunities for growth. This approach can serve as a model for trainees, helping them develop their own coaching skills. By developing trainees into coaches and promoting widespread faculty development around coaching competencies, GME programs can transform into organizations where faculty members are broadly prepared to capitalize on the potential of coaching in routine faculty-trainee and faculty-faculty interactions. Finally, by expanding this culture and skill development to include interprofessional colleagues, the DDO framework can be fully implemented within GME.

## Conclusion

To fully realize the potential of physicians-in-training, the push for coaching in medical education needs to go beyond just targeting individual coaching relationships to establishing a deliberately developmental culture. Coaching should become the shared responsibility of all individuals and deeply embedded within programs and organizations. Achieving this will necessitate significant organizational changes, such as prioritizing developmental aspirations at both the individual and organizational level, incorporating developmental practices in daily routines and, crucially, building developmental communities. Despite the challenges involved, such a transformation will be rewarded with a learning culture where trainees, faculty, and other health professionals are encouraged to challenge themselves, acknowledge mistakes, and reveal their authentic selves for continuous improvement.

## Additional File

The additional file for this article can be found as follows:

10.5334/pme.936.s1 Supplementary Table. Vignettes illustrating transformation of graduate medical education into a deliberately developmental organization at the mono, micro, meso and macro levels.
