# Supplementary material for: It Takes a Village: Optimal Graduate Medical Education Requires a Deliberately Developmental Organization
Source: Perspect Med Educ. 2023 Jul 28;12(1):282–93. doi: 10.5334/pme.936 (PMC10377742; doi:10.5334/pme.936)
Supplement: Supplementary Table. — Vignettes illustrating transformation of graduate medical education into a deliberately developmental organization at the mono, micro, meso and macro levels. [file pme-12-1-936-s1.pdf]

Supplementary Table: Vignettes illustrating transformation of graduate medical education into a deliberately developmental organization at the mono, micro, meso and macro levels*

Mono Level	*John is a first-year pediatric emergency medicine (PEM) fellow. After participating in a trauma and watching the third-year fellow in the role of trauma team leader (TTL), John self-reflects and identifies that he is not comfortable with assuming the TTL role. He decides to make becoming a strong TTL a goal (Developmental Aspirations).*<br><br>*Before his shift, John informs his attending of his goal (Developmental Communities). He shares that he feels his knowledge of trauma management is insufficient, and that he wonders if his personal disinclination towards public speaking is playing a factor. He asks to lead all trauma resuscitations during this shift (Developmental Practices).*
Micro Level	*During the shift, John assumes the role of TTL (Developmental Practices). When the surgical attending arrives, she agrees with John's plan and then assumes control of the trauma team. After the shift, John's attending – who knows John is working on leading traumas (Developmental Aspirations) – offers to help John identify what parts of his performance need improvement (Developmental Communities). He beings by applauding John for seizing the opportunity to lead the trauma before asking John to share what he was thinking while acting as TTL (Developmental Practices). Whatever John shares is received without judgment by his attending, who is explicit about his goal of helping John examine how to improve his performance.*<br><br>*The attending also began the shift by sharing his goal of improving his communication with nurses (Developmental Aspirations). While sitting in the charting area with all the trainees on his team, he asks each nurse to let him know whenever he fails to communicate something critical to him (Developmental Communities).*
Meso Level	<u>Division/Department:</u> *The PEM Division schedules shifts so that attendings and trainees arrive 15 minutes prior to assuming their clinical responsibilities and stay 15 minutes after their clinical responsibilities end (Developmental Practices). These 30 minutes of protected time are used for sharing of growth goals and feedback (Developmental Aspirations).*<br><br>*All PEM faculty and fellows receive training on coaching including conversational routines to encourage revealing internal thought processes and providing feedback (such as the R2C2 facilitated feedback model - build relationship, explore reactions, explore content and coach for performance change) (Developmental Communities).*<br><br>*The PEM Division's weekly conferences include protected time for PEM faculty and fellows to update and publicly share growth goals and to record them in an electronic platform (Developmental Aspirations). Faculty and fellows are expected to review each other's goals prior to working shifts together (Developmental Communities).*<br><br><u>Institution:</u> *Faculty reviews track and provide feedback on how faculty do at pursuing their own growth goals and at coaching others in doing the same (Developmental Aspirations). For example, faculty evaluations include questions on whether faculty are aware of trainees' growth goals, whether they provide feedback on these goals, and whether they share and receive feedback on their own areas for growth. Faculty are expected to complete additional training and/or clinical exposure in an area of weakness (Developmental Practices).*<br><br>*Faculty are expected to elicit and submit feedback from other health professionals related to growth goals. Other health professionals are given time and training to provide this feedback. Leaders from all health professions model seeking interprofessional feedback (Developmental Communities).*<br><br>*Professional development through constructive destabilization as well as receipt and incorporation of feedback are critical to promotion. Focus is not on the feedback received, but on actions taken to address it (Developmental Aspiration).*
Macro Level	*ACGME requires real-time sharing of competency-based assessments and tracking of implementation of learning goals by graduate medical education programs (Developmental Aspirations). Programs are given increased flexibility around training requirements to align clinical and didactic activities with identified trainee weaknesses (Developmental Practices). ACGME also requires programs to provide training in coaching (Developmental Communities).*<br><br>*The American Academy of Pediatrics requires setting learning goals (Developmental Aspirations) and creating an improvement plan for maintenance of certification (Developmental Practices). Proof of regular engagement in development of trainees and colleagues is also required for maintenance of certification (Developmental Communities).*

* Mono = individual, micro = individual coaching relationship, meso = division/department and/or institution, macro regulatory bodies
